# Neural crest Notch/Rbpj signaling regulates olfactory gliogenesis and neuronal migration†

**DOI:** 10.1002/dvg.23215

**Published:** 2018-08-08

**Authors:** Sophie R. Miller, Cristina Benito, Rhona Mirsky, Kristján R. Jessen, Clare V. H. Baker

**Affiliations:** ^1^ Department of Physiology, Development and Neuroscience University of Cambridge Cambridge CB2 3DY United Kingdom; ^2^ Department of Cell and Developmental Biology University College London, Gower Street London WC1E 6BT United Kingdom

**Keywords:** chicken embryo, gonadotropin‐releasing hormone (GnRH) neurons, mouse embryo, olfactory ensheathing glial cells

## Abstract

The neural crest‐derived ensheathing glial cells of the olfactory nerve (OECs) are unique in spanning both the peripheral and central nervous systems: they ensheathe bundles of axons projecting from olfactory receptor neurons in the nasal epithelium to their targets in the olfactory bulb. OECs are clinically relevant as a promising autologous cell transplantation therapy for promoting central nervous system repair. They are also important for fertility, being required for the migration of embryonic gonadotropin‐releasing hormone (GnRH) neurons from the olfactory placode along terminal nerve axons to the medial forebrain, which they enter caudal to the olfactory bulbs. Like Schwann cell precursors, OEC precursors associated with the developing olfactory nerve express the glial marker myelin protein zero and the key peripheral glial transcription factor Sox10. The transition from Schwann cell precursors to immature Schwann cells is accelerated by canonical Notch signaling via the Rbpj transcription factor. Here, we aimed to test the role of Notch/Rbpj signaling in developing OECs by blocking the pathway in both chicken and mouse. Our results suggest that Notch/Rbpj signaling prevents the cranial neural crest cells that colonize the olfactory nerve from differentiating as neurons, and at later stages contributes to the guidance of GnRH neurons.

## 1 INTRODUCTION

Olfactory ensheathing glial cells (OECs) are of considerable clinical interest since they can be cultured from biopsies of the nasal mucosa ‐ or, more invasively, from the olfactory bulb ‐ for autologous transplants that have shown promise in helping to promote central nervous system repair (see e.g., Barton, St John, Clarke, Wright, & Ekberg, 2017; Ekberg & St John, 2014; Ekberg, Amaya, Mackay‐Sim, & St John, 2012; Granger, Blamires, Franklin, & Jeffery, 2012; Granger, Franklin, & Jeffery, 2014; Kocsis, Lankford, Sasaki, & Radtke, 2009; Roet & Verhaagen, 2014; Watzlawick et al., 2016). Furthermore, OECs are important for fertility: they form the primary microenvironment for hypothalamic gonadotropin‐releasing hormone (GnRH) neurons during their embryonic migration from the olfactory placode to the forebrain (Geller, Kolasa, Tillet, Duittoz, & Vaudin, 2013; Geller et al., 2017), and defective OEC differentiation in the absence of the transcription factor Sox10 results in olfactory axon targeting defects and a significant reduction in the proportion of GnRH neurons that enter the forebrain (Barraud, St John, Stolt, Wegner, & Baker, 2013; Pingault et al., 2013). Loss‐of‐function *SOX10* mutations are found in roughly one‐third of cases of Kallmann's syndrome (combined anosmia and hypogonadotropic hypogonadism) with deafness (Pingault et al., 2013).

Apart from the requirement for Sox10 for normal OEC differentiation (Barraud et al., 2013; Pingault et al., 2013), the molecular mechanisms underlying OEC development have been relatively little studied, especially in comparison with those underlying the development of Schwann cells, the glia of all other peripheral nerves (reviewed by Jacob, 2015; Jessen, Mirsky, & Lloyd, 2015; Kastriti & Adameyko, 2017). Like all other peripheral glial cells (i.e., Schwann cells and the satellite glia of peripheral ganglia), whose differentiation also requires Sox10 (Britsch et al., 2001), OECs are derived from the embryonic neural crest (Barraud et al., 2010; Forni, Taylor‐Burds, Melvin, Williams, & Wray, 2011). The Sox10‐expressing 'Schwann cell precursors' associated with embryonic peripheral nerves can be distinguished from their neural crest progenitors by the expression of early glial markers such as myelin protein zero (Mpz, P0) and fatty acid‐binding protein 7 (brain lipid‐binding protein; brain fatty acid‐binding protein) (see Jacob, 2015; Jessen et al., 2015; Kastriti & Adameyko, 2017). Similarly, cells in the OEC lineage can first be identified in the chicken embryo via the onset of immunoreactivity for Mpz at embryonic day (E)3.5–4 (Drapkin & Silverman, 1999; Norgren, Ratner, & Brackenbury, 1992) and also by expression of *Sox10* (Barraud et al., 2010), in cells closely associated with the migrating neurons and axons of the olfactory nerve (Drapkin & Silverman, 1999; Norgren et al., 1992). In the mouse, developing OECs can first be identified at E10.5, as Sox10‐expressing cells associated with the 'migratory mass' of neurons and olfactory axons (Barraud et al., 2013; Forni et al., 2011).

Immature Schwann cells are both molecularly and phenotypically distinct from Schwann cell precursors: they express, for example, S100, glial fibrillary acidic protein (Gfap) and *Desert hedgehog* (*Dhh*), and are able to support their own survival via autocrine signaling (Dong et al., 1999; Meier et al., 1999), whereas Schwann cell precursors depend for their survival on axon‐associated neuregulin 1 type III (Dong et al., 1995). The canonical Notch/Rbpj signaling pathway (reviewed by Bray, 2016; Kovall, Gebelein, Sprinzak, & Kopan, 2017) promotes the transition from Schwann cell precursors to immature Schwann cells: the conditional deletion of either *Rbpj* or *Notch1* in Schwann cell precursors using a *Dhh‐Cre* line (Jaegle et al., 2003) delays this transition, while driving expression of the Notch intracellular domain to activate Notch signaling accelerates the transition (Woodhoo et al., 2009).

Here, we aimed to test the role of Notch/Rbpj signaling in developing OECs by using the Tol2 transposase/‘Tet‐on' electroporation system (Sato et al., 2007; Watanabe et al., 2007) to insert a doxycycline‐inducible dominant negative *Rbpj* construct (Kohyama et al., 2005; Sato et al., 2008) into the genome of chicken cranial neural crest cells, and by using an *Mpz‐Cre* driver line (Feltri et al., 1999, 2002) combined with an *Rbpj^flox/flox^* line (Tanigaki et al., 2002) to delete *Rbpj* in mouse OECs. Our chicken experiments showed that Notch/Rbpj signaling is required in the cranial neural crest‐derived cells that colonize the olfactory nerve to prevent them from differentiating as neurons, while our mouse experiments revealed defects in GnRH neuron localization when Notch/Rbpj signaling was blocked in OECs.

## 2 RESULTS

### 2.1 Blocking Notch/Rbpj signaling in cranial neural crest‐derived cells with temporal control

In order to block Notch/Rbpj signaling in cranial neural crest‐derived cells with temporal control, we used the Tol2 transposase/“Tet‐on” electroporation system (Sato et al., 2007; Watanabe et al., 2007) to integrate doxycycline‐inducible constructs into the genome of cranial neural crest cells. To achieve maximum electroporation efficiency, we performed electroporation *ex ovo* and then grafted targeted midbrain and caudal forebrain neural folds (containing the premigratory neural crest cells that contribute to the frontonasal mesenchyme and OECs; Barraud et al., 2010) into untargeted host embryos.

**Figure 1:**
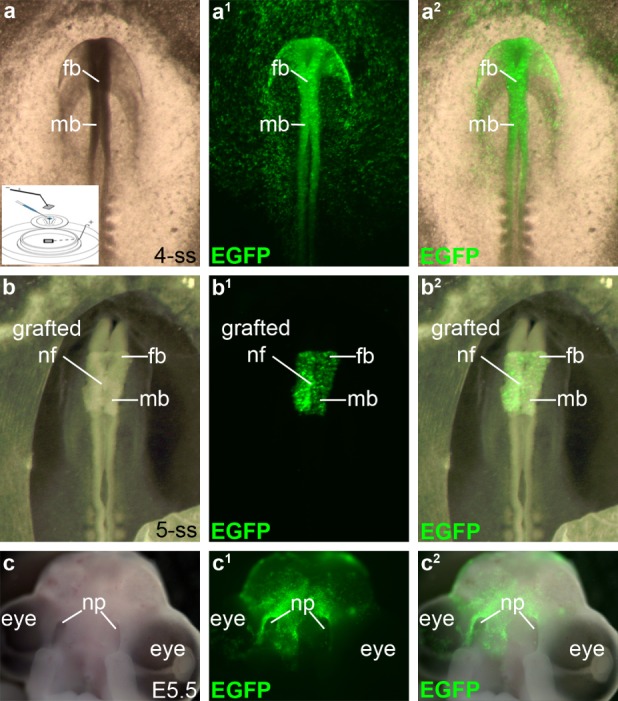
*Ex ovo* electroporation and neural fold grafting to target cranial neural crest precursors. (**a–a^2^**) An *EGFP*‐targeted embryo that was electroporated *ex ovo* at HH5 (head process stage) and cultured in EC culture (Chapman et al., 2001) to reach the 4 somite‐stage (HH8). Almost the entire cranial ectoderm expresses EGFP, including the cranial neural folds containing premigratory neural crest cells. Inset shows a schematic of the *ex ovo* electroporation procedure [modified from “Electroporation for early chick embryos using New's culture (gastrula), Application note 1”; http://www.sonidel.com/sonidel/in-ovo-electroporation/]. For a detailed description, see Section 4. (**b–b^2^**) A 5 somite‐stage (HH8+) embryo *in ovo*, photographed immediately after receiving a bilateral neural fold graft (at the level of the caudal forebrain and midbrain) from an *EGFP*‐targeted donor embryo. (**c–c^2^**) Ventral view of an embryo that had been similarly grafted with *DN‐Rbpj*/*EGFP*‐targeted donor neural folds and allowed to develop until E5.5, two days after doxycycline injection at E3.5. EGFP‐positive neural crest‐derived cells are present in the frontonasal process, including near the nasal pits. E, embryonic day; fb, forebrain; mb, midbrain; nf, neural folds; np, nasal pits; ss, somite‐stage

Donor embryos were explanted onto filter paper at head process to head‐fold stages (Hamburger‐Hamilton stages [HH]5‐6; 19–25 hours of incubation; Hamburger & Hamilton, 1951) and the entire cranial ectoderm co‐electroporated with a mixture of three plasmids: *pCAGGS‐T2TP* (Kawakami & Noda, 2004; Sato et al., 2007), encoding Tol2‐transposase under the control of the synthetic CAGGS promoter (Niwa, Yamamura, & Miyazaki, 1991); the Tol2‐integratable *pT2K‐CAGGS‐rtTA2^S^ M2* and construct (Sato et al., 2007), encoding the reverse (“Tet‐on”) tetracycline transactivator protein variant rtTA2^S^M2 (Urlinger et al., 2000); and either the Tol2‐integratable *pT2K‐DN‐Rbpj‐BI‐EGFP* construct (Kohyama et al., 2005; Sato et al., 2008), encoding a dominant negative form of the mouse transcription factor Rbpj (R218H, which will compete with endogenous Rbpj to bind the Notch intracellular domain, but which cannot bind DNA; Chung, Hamaguchi, Honjo, & Kawaichi, 1994; Kato et al., 1997) plus EGFP (bidirectional transcription controlled by a single tetracycline‐response element), or the Tol2‐integratable control construct *pT2K‐CAGGS‐EGFP*, encoding EGFP alone (Sato et al., 2007) (Figure 1a, inset).

The electroporated embryos were cultured according to the Early Chick (EC) culture method (Chapman, Collignon, Schoenwolf, & Lumsden, 2001) for 9–10 hours until cranial neural folds had developed (roughly 2–5 somites; HH7+ to 8+), but before neural crest emigration had started (midbrain‐level neural crest cells are the first to emerge, from the 6‐somite stage; Tosney, 1982) (Figure 1a–a^2^). Midbrain and caudal forebrain neural folds were grafted bilaterally from electroporated donor embryos into wild‐type hosts *in ovo* (Figure 1b–b^2^) to ensure that, upon final analysis, only donor cranial neural fold‐derived cells, including neural crest cells, expressed the Tol2‐integrated constructs. To initiate expression of *DN‐Rbpj*/*EGFP* (and thus block Notch/Rbpj signaling), the host eggs were injected with doxycycline at embryonic day (E)3.5, when chicken OEC precursors can be detected via the onset of immunoreactivity on the olfactory nerve for the early glial marker myelin protein zero (Mpz, P0) (Drapkin & Silverman, 1999). (The control *EGFP* construct is constitutively expressed.) Embryos were collected for analysis two days after doxycycline injection, at E5.5 (Figure 1c–c^2^).

### 2.2 When Notch/Rbpj signaling is blocked from E3.5 in cranial neural crest‐derived cells, most targeted cells on the olfactory nerve form neurons

**Figure 2:**
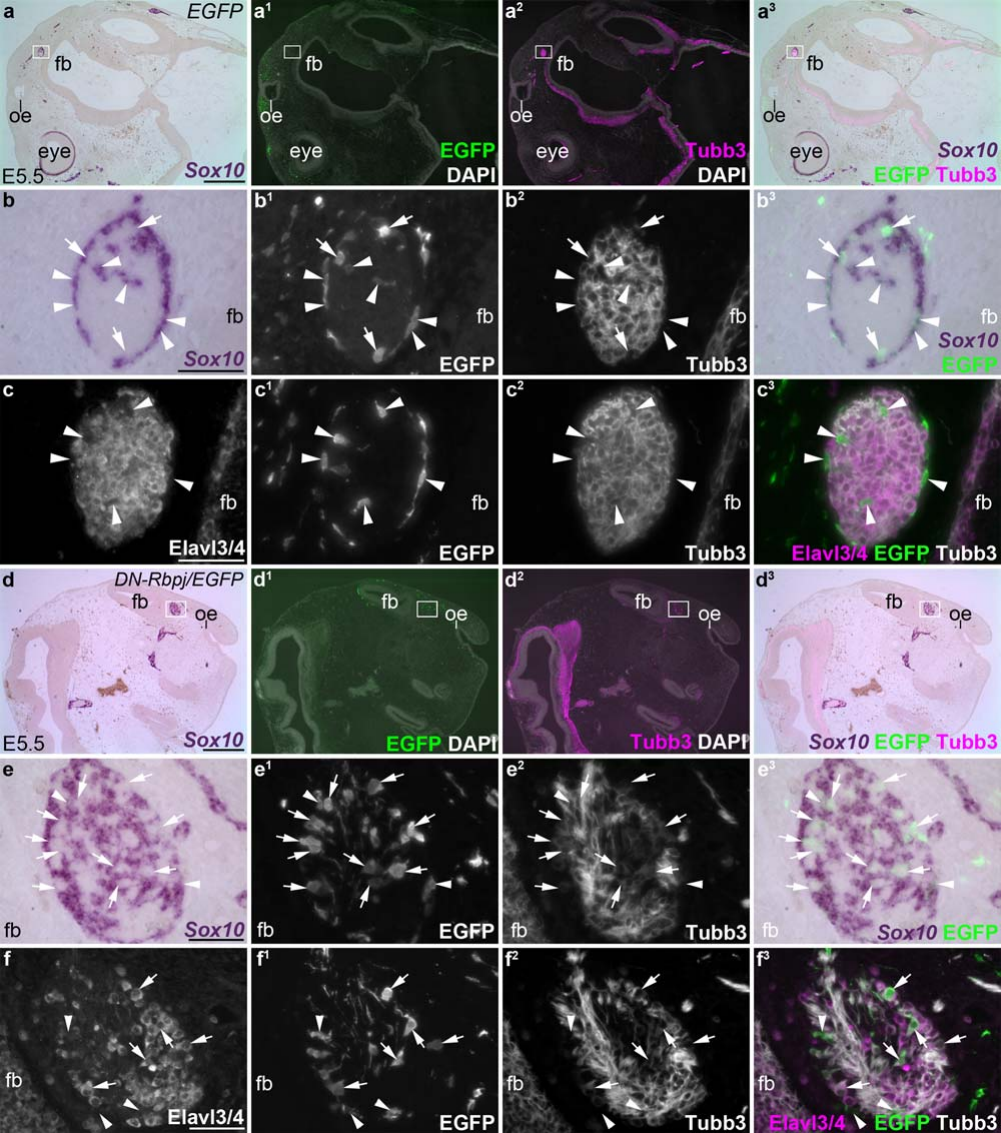
Blocking Notch/Rbpj signaling in cranial neural crest‐derived cells from E3.5 results in neurogenesis on the olfactory nerve. Parasagittal sections of chicken embryos at E5.5 that had received grafts of midbrain and caudal forebrain neural folds from *ex ovo*‐electroporated donor embryos at E1.5, with doxycycline injection at E3.5. (**a–a^3^**) Control *EGFP*‐targeted embryo, showing ISH for *Sox10* and immunostaining for EGFP and Tubb3 (neuronal class III beta‐tubulin). *EGFP*‐targeted cells are seen in the neural folds, frontonasal mass and on the olfactory nerve. (**b–**b^3^) Higher‐power view of boxed region in panels a–a^3^, showing the olfactory nerve near the forebrain. A few *EGFP*‐targeted cells on the olfactory nerve lack expression of the OEC marker *Sox10* (arrows), but most are *Sox10*‐positive (arrowheads highlight examples). (**c–**c^3^) Immunostaining on a nearby section for the neuron‐specific marker Elavl3/4 shows that *EGFP*‐targeted neural crest‐derived cells on the olfactory nerve are Elavl3/4‐negative, i.e., are not neurons (arrowheads highlight examples). (**d–d^3^**) A *DN‐Rbpj*/*EGFP*‐targeted embryo (the same embryo shown in Figure 1c–c^2^), showing ISH for *Sox10* and immunostaining for EGFP and Tubb3. *DN‐Rbpj*/*EGFP*‐targeted cells are seen in the frontonasal mass and along the olfactory nerve. (**e–**e^3^) Higher‐power view of boxed region in panels d‐d^3^, showing the olfactory nerve near the forebrain. A few *DN‐Rbpj*/*EGFP*‐targeted cells on the olfactory nerve express *Sox10* (arrowheads highlight examples), but most are *Sox10*‐negative (arrows highlight examples). (**f–**f^3^) Immunostaining on a nearby section for the neuron‐specific marker Elavl3/4 shows that although some *DN‐Rbpj*/*EGFP*‐targeted neural crest‐derived cells on the olfactory nerve are not neurons (arrowheads), many are neurons (arrows highlight examples). E, embryonic day; fb, forebrain; oe, olfactory epithelium. Scale‐bars: 100 μm (a–a^3^, d–d^3^), 50 μm (b–c^3^, e–f^3^)

**Figure 3:**
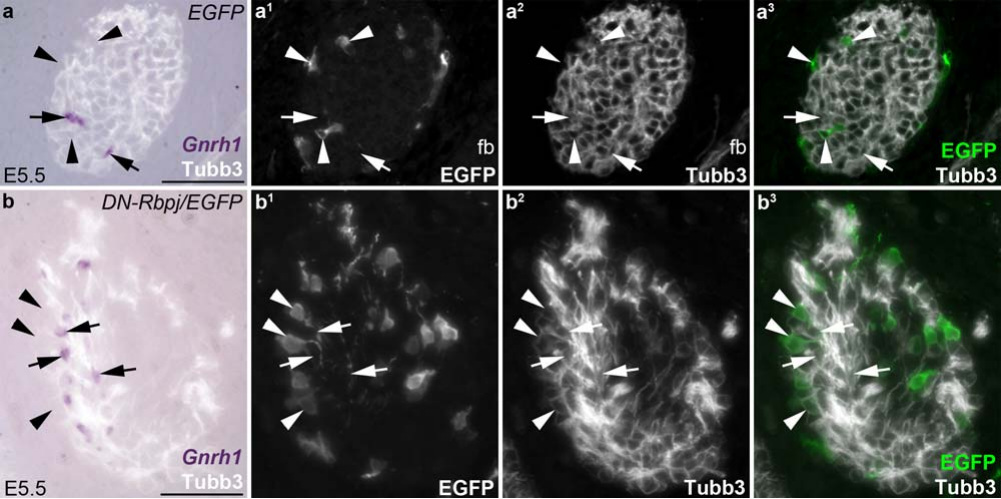
The neural crest‐derived neurons on the olfactory nerve that form after blocking Notch/Rbpj signaling are distinct from GnRH neurons. Parasagittal sections of chicken embryos at E5.5 that had received grafts of midbrain and caudal forebrain neural folds from *ex ovo*‐electroporated donor embryos at E1.5, with doxycycline injection at E3.5. (**a–**a^3^) The olfactory nerve near the forebrain from the same series of sections through the control *EGFP*‐targeted embryo shown in Figure 2a–c^3^, after ISH for *Gnrh1* and immunostaining for EGFP and Tubb3 (neuronal class III beta‐tubulin). *EGFP*‐targeted cells on the olfactory nerve (arrowheads highlight examples) do not express *Gnrh1* (arrows highlight examples of GnRH neurons). (**b–**b^3^) The olfactory nerve near the forebrain from the same series of sections through the *DN‐Rbpj*/*EGFP*‐targeted embryo shown in Figure 2d–f^3^, showing ISH for *Gnrh1* and immunostaining for EGFP and Tubb3. *DN‐Rbpj*/*EGFP*‐targeted cells on the olfactory nerve (arrowheads highlight examples) do not express *Gnrh1* (arrows highlight examples of GnRH neurons). E, embryonic day; fb, forebrain; oe, olfactory epithelium. Scale‐bars: 50 μm

Two days after doxycycline injection, at E5.5 (*n* = 2), most control *EGFP*‐targeted neural crest‐derived cells on the olfactory nerve expressed the OEC marker *Sox10*, as expected (Barraud et al., 2010) (Figure 2a–b^3^). [The presence of *Sox10*‐negative *EGFP*‐targeted cells could reflect the formation of non‐glial neural crest derivatives, such as endoneurial fibroblasts (Joseph et al., 2004) and/or perivascular cells, which are neural crest‐derived in the region of the forebrain (Etchevers, Vincent, Le Douarin, & Couly, 2001); indeed, the perivascular cell marker *Pdgfrb* is detectable in some cells on the olfactory nerve at E6.5 (Miller, Perera, & Baker, 2017).] Immunostaining for the neuronal RNA‐binding proteins Elavl3/Elavl4 (HuC/D, hereafter Elavl3/4; Okano & Darnell, 1997; Pascale, Amadio, & Quattrone, 2008) revealed many Elavl3/4‐positive neuron cell bodies on the olfactory nerve, but, as expected (Barraud et al., 2010), *EGFP*‐targeted neural crest‐derived cells were not neurons (Figure 2c–c^3^). In contrast, many of the *DN‐Rbpj*/*EGFP*‐targeted neural crest‐derived cells on the olfactory nerve at E5.5 (*n* = 4) were *Sox10*‐negative (Figure 2d–e^3^) and expressed Elavl3/4, i.e., were neurons (Figure 2f‐f^3^). These neurons were distinct from GnRH neurons (Figure 3a‐b^3^). Cell counting revealed that 72% of *DN‐Rbpj*/*EGFP*‐targeted cells on the olfactory nerve at E5.5 were Elavl3/4‐positive neurons (*n* = 474 *DN‐Rbpj*/*EGFP*‐targeted cells counted across both olfactory nerves from two embryos).

Overall, these data show that blocking Notch/Rbpj signaling in cranial neural crest‐derived cells for two days from E3.5 (when Mpz‐positive cells in the OEC lineage can first be identified on the olfactory nerve; Drapkin & Silverman, 1999) resulted in a majority of the targeted cells on the olfactory nerve adopting a neuronal fate (distinct from migrating GnRH neurons, which are olfactory placode‐derived; Barraud et al., 2010; Sabado, Barraud, Baker, & Streit, 2012). This suggests that Notch/Rbpj signaling is required to maintain the glial identity of developing OECs, at least from E3.5 to E5.5.

### 2.3 When Notch/Rbpj signaling is blocked in mouse OECs, more GnRH neurons are found in the lateral regions of the olfactory bulbs

We also investigated the effect of blocking Notch/Rbpj signaling on mouse OEC development, by analyzing E16.5 mouse embryos from crosses between the transgenic *Mpz‐Cre* (*myelin protein zero‐Cre*; *P0‐Cre*) driver line (Feltri et al., 1999, 2002) and homozygous *Rbpj^flox/flox^* mice (Tanigaki et al., 2002). Rat OECs express *Mpz* from E13 through to adulthood (Lee et al., 2001). We did not find any evidence for ectopic neurogenesis on the olfactory nerve in *Mpz‐Cre*;*Rbpj^flox/flox^* mutant mouse embryos at E16.5 (data not shown). However, Cre recombination in the Schwann cell lineage in *Mpz‐Cre* mouse embryos does not occur until between E13.5‐E14.5 (Yu et al., 2005; Woodhoo et al., 2009), when Schwann cell precursors are transitioning to immature Schwann cells (Dong et al., 1999). This is considerably later than the onset of *Mpz* expression in Schwann cell precursors (Jessen et al., 2015). This could explain why we did not detect the neurogenesis phenotype observed in our chicken experiments.

**Figure 4:**
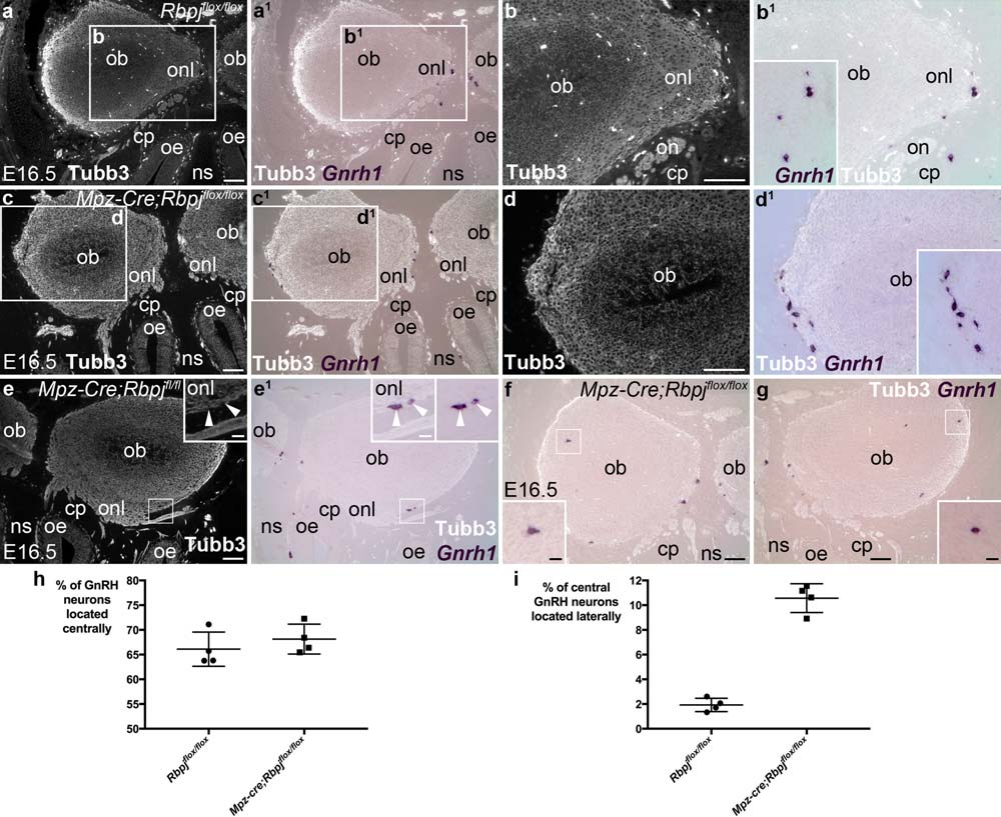
In *Mpz‐Cre;Rbpj^flox/flox^* mouse embryos at E16.5, more GnRH neurons are found laterally in the olfactory bulbs. Coronal sections through the olfactory system of E16.5 control *Rbpj^flox/flox^* and mutant *Mpz‐Cre;Rbpj^flox/flox^* littermates, showing ISH for *Gnrh1* to detect GnRH neurons and immunostaining for Tubb3. (**a,a^1^**) In a control *Rbpj^flox/flox^* mouse embryo, GnRH neurons are located at the medial surfaces of the olfactory bulbs. (**b,b^1^**) Higher‐power view of boxed region in a,a^1^; inset shows *Gnrh1* alone. (**c,c^1^**) In a *Mpz‐Cre;Rbpj^flox/flox^* mutant litter‐mate, GnRH neurons are located at the lateral edge of the olfactory bulb, as well as medially. (**d,d^1^**) Higher‐power view of boxed region in c,c^1^; inset shows *Gnrh1* alone. (**e,e^1^**) In a different *Mpz‐Cre;Rbpj^flox/flox^* embryo, some GnRH neurons (arrowheads in insets, which show a higher‐power view of the boxed region) are also seen in the lateral olfactory nerve layer. The two insets in panel e^1^ show the same image with and without Tubb3. (**f,g**) Right and left olfactory bulbs from a third *Mpz‐Cre;Rbpj^flox/flox^* embryo, showing a few GnRH neurons scattered laterally in both olfactory bulbs (insets show higher‐power views of boxed regions), as well as medially. (**h**) Scatter plot (bars show mean and s.d.) showing the percentage per embryo of all *Gnrh1*‐positive cells counted that were located centrally at E16.5, in control *Rbpj^flox/flox^* embryos (mean 66.1 ± 3.5%; *n* = 4 embryos from 2 litters; 276–350 GnRH neurons counted per embryo) versus *Mpz‐Cre;Rbpj^flox/flox^* mutant embryos (mean 68.1 ± 1.5%; *n* = 4 embryos from 2 litters; 241–357 GnRH neurons counted per embryo). The difference between the means is not significant (*p* = 0.411; unpaired two‐tailed Student's *t*‐test; *t* = 0.883, 6 degrees of freedom). (**i**) Scatter plot (bars show mean and s.d.) showing the percentage per embryo of all centrally located *Gnrh1*‐positive cells that were found laterally at E16.5, in control *Rbpj^flox/flox^* embryos (mean 1.9 ± 0.5%; *n* = 4 embryos from 2 litters; 176–230 central GnRH neurons counted per embryo; 3–6 of these per embryo were located laterally) versus *Mpz‐Cre;Rbpj^flox/flox^* mutant embryos (mean 10.6 ± 1.2%; *n* = 4 embryos from 2 litters; 160–258 central GnRH neurons counted per embryo; 17–23 of these per embryo were located laterally). The mean is ∼5.5‐fold higher for *Mpz‐Cre;Rbpj^flox/flox^* mutants (*p* < 0.0001; unpaired two‐tailed Student's *t*‐test; *t* = 13.44, 6 degrees of freedom). cp, cribriform plate; E, embryonic day; ns, nasal septum; ob, olfactory bulb; oe, olfactory epithelium; on, olfactory nerve; onl, olfactory nerve layer. Scale‐bars: 100 μm in all panels except insets in e–g: 20μm

Nevertheless, our analysis of *Mpz‐Cre*;*Rbpj^flox/flox^* mutant embryos at E16.5 revealed an interesting defect in GnRH neuron localization. In *Rbpj^flox/flox^* embryos at E16.5, almost all centrally located *Gnrh1*‐positive neurons were detected on the medial surfaces of the olfactory bulbs (Figure 4a‐b^1^), as expected, i.e., migrating along terminal nerve axons coursing along the medial olfactory bulb towards their more caudal entry point in the rostral forebrain (Schwanzel‐Fukuda & Pfaff, 1989; Taroc, Prasad, Lin, & Forni, 2017; Yoshida, Tobet, Crandall, Jimenez, & Schwarting, 1995). However, in *Mpz‐Cre;Rbpj^flox/flox^* litter‐mates, we noticed that GnRH neurons were also located at the lateral surfaces of the olfactory bulbs (Figure 4c‐g). Cell counting revealed that there was no difference at E16.5 in the mean percentage per embryo (± s.d.) of all GnRH neurons that were located centrally (Figure 4h): 66.1 ± 3.5% for *Rbpj^flox/flox^* embryos (*n* = 4 from 2 litters; 276–350 GnRH neurons counted per embryo), versus 68.1 ± 3.0% for *Mpz‐Cre;Rbpj^flox/flox^* litter‐mates (*n* = 4 from 2 litters; 241–357 GnRH neurons counted per embryo). However, there was an approximately five‐fold increase in the mean percentage per embryo (± s.d.) of central GnRH neurons that were located laterally at E16.5 (Figure 4i): from 1.9 ± 0.5% in *Rbpj^flox/flox^* embryos (*n* = 4 embryos from 2 litters; 176–230 central GnRH neurons counted per embryo; 3–6 of these per embryo were located laterally) to 10.6 ± 1.2% in *Mpz‐Cre;Rbpj^flox/flox^* embryos (*n* = 4 embryos from two litters; 160–258 central GnRH neurons counted per embryo; 17–23 of these per embryo were located laterally). Comparison of the means using an unpaired two‐tailed Student's *t*‐test showed this difference to be highly statistically significant (*p* < 0.0001; Figure 4i).

Overall, our data suggest that at early stages of OEC development, Notch/Rbpj signaling is required to block adoption of a neuronal fate, while at later stages, activation of this pathway contributes to the guidance of GnRH neurons.

## 3 DISCUSSION

### 3.1 Notch/Rbpj signaling prevents neural crest‐derived cells on the olfactory nerve from differentiating as neurons

During chicken olfactory system development, the Sox10‐expressing ensheathing glia of the olfactory nerve originate from the cranial neural crest (Barraud et al., 2010), while the neurons on the olfactory nerve, including GnRH neurons, are derived from the olfactory placode (Barraud et al., 2010; Sabado et al., 2012). Here, we investigated the role of Notch/Rbpj signaling in developing chicken OECs by using the Tol2 transposase/‘Tet‐on’ electroporation system (Sato et al., 2007; Watanabe et al., 2007) to insert a doxycycline‐inducible construct encoding a dominant negative form of mouse Rbpj (Chung et al., 1994; Kato et al., 1997) into the genome of cranial neural crest cell precursors *ex ovo* (to maximize targeting efficiency), followed by *in ovo* grafting of targeted midbrain and caudal forebrain neural folds into unmanipulated host embryos. We found that inhibiting Notch/Rbpj signaling for two days in cranial neural crest‐derived cells from E3.5 (when Mpz‐positive cells in the OEC lineage can first be identified on the olfactory nerve; Drapkin & Silverman, 1999) promoted the adoption of a neuronal fate (distinct from migrating olfactory placode‐derived GnRH neurons; Barraud et al., 2010; Sabado et al., 2012) by around 70% of targeted cells on the olfactory nerve (although other targeted cells retained *Sox10* expression, perhaps because they had not yet down‐regulated *Sox10*, or owing to varying levels of transgene expression).

These results suggest that Notch/Rbpj signaling in the cranial neural crest‐derived frontonasal mesenchyme cells that colonize the olfactory nerve is required, at least between E3.5 and E5.5, to prevent them from adopting a neuronal fate on the olfactory nerve. Neural crest cells in the frontonasal mass originate from the rostral midbrain and caudal forebrain; these neural crest cells migrate rostrally to populate the frontonasal mass (Noden, 1975). This population of neural crest cells does not normally form neurons (Narayanan & Narayanan, 1978; Noden, 1975), but when grafted adjacent to the rostral hindbrain, will contribute neurons to the trigeminal ganglion (Baker, Bronner‐Fraser, Le Douarin, & Teillet, 1997; Noden, 1975). Taken together, these results suggest that Notch/Rbpj signaling is required to prevent neuronal differentiation by the neural crest‐derived cells that colonize the olfactory nerve.

In contrast to the results obtained in chicken, we did not detect ectopic neurogenesis on the mouse olfactory nerve at E16.5 when Notch/Rbpj signaling was abrogated in peripheral glial cells, by deleting *Rbpj* using the *Mpz‐Cre* driver line (Feltri et al., 1999, 2002; Tanigaki et al., 2002). This driver line has been used to disrupt various genes in the Schwann cell lineage (e.g., Bolino et al., 2004; D'Antonio et al., 2006; Feltri et al., 2002; Yu et al., 2005), including *Rbpj* (Woodhoo et al., 2009). However, these studies suggested that Cre‐mediated recombination is not initiated until at least between E13.5 and E14.5 (Woodhoo et al., 2009; Yu et al., 2005), when Schwann cell precursors are transitioning to immature Schwann cells (Dong et al., 1999). In the mouse, Sox10‐positive OEC precursors can already be detected in close association with the neurons and axons emerging from the olfactory placode at E10.5 (Barraud et al., 2013; Forni et al., 2011). Thus, the loss of Notch/Rbpj signaling in *Mpz‐Cre;Rbpj^flox/flox^* embryos may be too late to affect OEC precursor differentiation. This would explain the difference with the chicken phenotype, where Notch/Rbpj signaling was blocked from E3.5, when Mpz‐positive OEC precursors can first be detected (Drapkin & Silverman, 1999).

### 3.2 OEC precursors are multipotent

Previously, we used the Tol2 transposase/‘Tet‐on’ *in ovo* electroporation system (Sato et al., 2007; Watanabe et al., 2007) to drive *NotchΔE*, encoding a constitutively active form of mouse Notch1 (Kopan, Schroeter, Weintraub, & Nye, 1996; Sato et al., 2008), in cranial neural crest‐derived cells from E4 (Miller et al., 2017). This proved to be sufficient to convert both frontonasal mesenchyme cells, and perhaps also developing OECs, to a different cranial neural crest cell fate, namely *Pdgfrb*‐positive perivascular cells (Miller et al., 2017). Here, we found that most targeted cranial neural crest‐derived cells on the olfactory nerve formed neurons when Notch/Rbpj signaling was blocked from E3.5. The competence of OEC precursors to adopt different fates in response to Notch signaling activation or repression is at least partly reminiscent of the multipotent 'Schwann cell precursors' found on other peripheral nerves: during normal development, these form not only immature Schwann cells but also endoneurial fibroblasts (Joseph et al., 2004), melanocytes (Adameyko et al., 2009), odontoblasts (Kaukua et al., 2014), parasympathetic neurons (Dyachuk et al., 2014; Espinosa‐Medina et al., 2014), enteric neurons (Espinosa‐Medina et al., 2017; Uesaka, Nagashimada, & Enomoto, 2015) and adrenal chromaffin cells (Furlan et al., 2017).

### 3.3 Notch/Rbpj signaling in embryonic OECs contributes to GnRH neuron guidance

In rodents, hypothalamic GnRH neurons migrate along terminal nerve axons, which segregate as a ventro‐caudally oriented branch from the vomeronasal nerve that projects along the medial olfactory bulbs and enters the medial forebrain caudal to the olfactory bulbs, terminating in septal and preoptic areas (Geller et al., 2013; Schwanzel‐Fukuda & Pfaff, 1989; Schwanzel‐Fukuda, 1999; Taroc et al., 2017; Yoshida et al., 1995). The terminal nerve is a plexiform, ganglionated nerve containing distinct subpopulations of neurons, including GnRH neurons and neuropeptide Y‐immunoreactive neurons (for reviews, see e.g. Demski, 1993; Larsell, 1950; Ma, Fleischer, Breer, & Eisthen, 2015; Schwanzel‐Fukuda, 1999; von Bartheld, 2004; Wirsig‐Wiechmann, Wiechmann, & Eisthen, 2002). The terminal nerve is thought to be neuromodulatory on the olfactory epithelium and, at least in teleost fish, on the retina (for a comprehensive recent review of functional studies, see Ma et al., 2015). GnRH neurons persist into adulthood along the course of the terminal nerve, including in the largest of the terminal nerve ganglia, the 'ganglion terminale' located on the medio‐caudal aspect of the olfactory bulb (Schwanzel‐Fukuda, 1999).

In the mouse embryo, OECs are intimately associated with GnRH neurons throughout their migration (Geller et al., 2013, 2017). We and others previously showed that defective OEC differentiation in *Sox10*‐null mice results in a significant reduction in the proportion of GnRH neurons entering the forebrain, as well as defects in olfactory axon targeting (Barraud et al., 2013; Pingault et al., 2013). Here, we found that deleting *Rbpj* in OECs using an *Mpz‐Cre* driver line (Feltri et al., 1999, 2002; Tanigaki et al., 2002) had no effect on the proportion of GnRH neurons entering the forebrain, but led to a roughly five‐fold increase in the mean percentage of GnRH neurons located in lateral rather than medial regions of the olfactory nerve layer and olfactory bulbs at E16.5 (from 1.9 ± 0.5% to 10.6 ± 1.2% of all centrally located GnRH neurons counted).

A recent study using GnRH immunostaining and 3D imaging of solvent‐cleared organs (3DISCO; Ertürk et al., 2012) described a single‐cell‐thick “ring” of GnRH neurons around the olfactory bulbs in both human and E16 mouse embryos (Casoni et al., 2016). In adult mice, terminal nerve GnRH neurons were previously described as including “an arborizing network of cell bodies at the level of the central portion of the olfactory bulb”, in connection with the ganglion terminale (Jennes, 1986). It is plausible that the few *Gnrh1*‐positive cells we identified on sections in the lateral olfactory bulbs of control embryos at E16.5 are part of this “ring”. The roughly five‐fold increase in the number of *Gnrh1*‐positive cells located laterally in Mpz‐*Cre;Rbpj^flox/flox^* embryos may suggest that Notch/Rbpj signaling in OECs somehow helps to prevent more GnRH neurons from migrating laterally around the olfactory bulbs to join this “ring”. In this context, it is interesting that terminal nerve axons and olfactory/vomeronasal axons express different guidance cue receptors (Taroc et al., 2017), and that OECs located in different regions of the main olfactory system (peripheral olfactory nerve, outer olfactory nerve layer, inner olfactory nerve layer) show at least some molecular and phenotypic heterogeneity (reviewed by Ekberg et al., 2012; Ekberg & St John, 2015). It is possible, therefore, that molecular differences between terminal nerve OECs and olfactory/vomeronasal nerve OECs ‐ potentially involving Notch/Rbpj signaling ‐ somehow contribute to embryonic GnRH neuron guidance, restricting most GnRH neurons to the medial pathway. Testing this speculative hypothesis must await a more detailed spatiotemporal characterization of OECs at the molecular level.

### 3.4 Conclusions

Overall, we conclude that Notch/Rbpj signaling plays different roles during OEC development. Our chicken electroporation data suggest that Notch/Rbpj signaling prevents neuronal differentiation by the cranial neural crest‐derived cells that colonize the olfactory nerve, while our conditional mouse mutant analysis suggests that Notch/Rbpj signaling in OECs later helps to restrict most migrating GnRH neurons to medially coursing terminal nerve axons.

## 4 MATERIALS AND METHODS

### 4.1 Embryos

Fertilized chicken (*Gallus gallus domesticus*) eggs were obtained from Winter Egg Farm (Royston, Hertfordshire, UK). All work with chicken embryos was conducted in accordance with the UK Animals (Scientific Procedures) Act 1986. Experiments using *Rbpj^flox/flox^* mice (Tanigaki et al., 2002) and *Mpz‐Cre* (*P0‐Cre*) mice (Feltri et al., 1999, 2002) were conducted in accordance with the UK Animals (Scientific Procedures) Act 1986, with appropriate project and personal licenses in place.

### 4.2 Electroporation constructs

Electroporation constructs were kind gifts of Yoshiko Takahashi (Kyoto University, Kyoto, Japan) and Hideyuki Okano (Keio University, Tokyo, Japan): (a) *pCAGGS‐T2TP* (Kawakami & Noda, 2004; Sato et al., 2007), encoding Tol2 transposase driven by the synthetic CAGGS promoter (Niwa et al., 1991); (b) *pT2K‐CAGGS‐rtTA2^S^M2* (Sato et al., 2007), a Tol2‐integratable construct encoding the reverse (“Tet‐on”) tetracycline transactivator protein variant rtTA2^S^M2 (Urlinger et al., 2000); (c) *pT2K‐DN‐Rbpj‐BI‐EGFP* (Chung et al., 1994; Kohyama et al., 2005; Sato et al., 2008), a Tol2‐integratable, tetracycline‐dependent construct encoding a dominant negative version of the mouse transcription factor Rbpj (mutation R218H, which decreases DNA binding activity to 2% of wild‐type; Chung et al., 1994) plus EGFP (bidirectional transcription controlled by a single tetracycline‐response element); (d) *pT2K‐CAGGS‐EGFP* (Sato et al., 2007), a Tol2‐integratable construct encoding EGFP alone. The Qiagen EndoFree Plasmid Maxi kit was used to prepare all constructs at a stock concentration of 5 μg/μl.

### 4.3 Chicken embryo *ex ovo* electroporation, *in ovo* grafting, fixation and histology

Fertilized chicken eggs were incubated in a humidified atmosphere at 38°C for 22–24 hours until HH5–6 (head process to head fold stages). Half were set aside to be the host embryos for neural fold grafts. The remaining embryos were explanted using a Whatman filter paper ring as described (Chapman et al., 2001) and placed in an electroporation chamber (Voiculescu, Papanayotou, & Stern, 2008) containing simple saline solution, with the positive electrode forming the base of the chamber. A 1:1:1 mix of *pCAGGS‐T2TP*, *pT2K‐CAGGS‐rtTA2^S^M2* and either *pT2K‐DN‐Rbpj‐BI‐EGFP* or control *pT2K‐CAGGS‐EGFP*, at a final concentration of 1.4 μg/μl each, mixed with Fast Green to a final dilution of 2% and sucrose to a final concentration of 8%, was micro‐pipetted over the cranial ectoderm and a “plate”‐type negative electrode positioned over the embryo (Figure 1a inset). A Pulse Generator CUY21EX electroporator (BEX Co., Ltd., Japan) was used to apply across the entire blastoderm an initial burst of 50V and five subsequent pulses of 8 V for 75 milliseconds at 50 millisecond intervals, leading to highly efficient targeting of the ectoderm (Figure 1a^1,^a^2^). Each electroporated embryo was placed onto an agar‐albumen plate according to the EC culture method (Chapman et al., 2001) and returned to the incubator, together with the remaining unopened eggs, for a further 9–10 hours to reach 2–5 somites. The remaining eggs were opened and 1% black ink (Fount India, Pelikan) in filtered phosphate‐buffered saline (PBS) was injected underneath the blastoderm to visualize the embryo. Electroporated donor and host embryos were not always at precisely the same stage. The neural folds encompassing the caudal forebrain and midbrain were dissected bilaterally from a wild‐type host embryo using a pulled glass needle and replaced with the equivalent region from an electroporated donor embryo (Figure 1b–b^2^). The window was sealed with Parafilm and the egg returned to the incubator. At E3.5, the Parafilm was removed and 500 µl of 100 μg/μl doxycycline (Clontech) injected under the embryo (as described by Sato et al., 2007). The eggs were sealed with Parafilm and returned to the incubator for 2 days before collection and analysis (Figure 1c‐c^2^). Surviving embryos were fixed in modified Carnoy's fixative (6 volumes ethanol, 3 volumes 37% formaldehyde, 1 volume glacial acetic acid), dehydrated into ethanol, cleared in Histosol (National Diagnostics) and embedded in paraffin wax for sectioning at 6 μm on a rotary microtome (Microm).

### 4.4 Mouse embryo fixation and histology

Embryos were decapitated and immersion‐fixed overnight in 4% paraformaldehyde in PBS at 4°C. They were cryoprotected by incubating overnight at 4°C in 30% sucrose in diethylpyrocarbonate‐treated PBS, embedded in O.C.T. compound (Tissue Tek) and flash‐frozen in isopentane on dry ice. Ten micrometer sections were taken on a rotary cryostat (Bright Instrument Company).

### 4.5 *In situ* hybridization and immunohistochemistry on sections

Chicken *Sox10* (Cheng, Cheung, Abu‐Elmagd, Orme, & Scotting, 2000) was a kind gift of Marianne Bronner (Caltech, Pasadena, USA). Mouse *Gnrh1* was previously cloned as described (Barraud et al., 2013). Digoxigenin‐labeled antisense riboprobes were generated as described (Henrique et al., 1995) and *in situ* hybridization on sections, followed by immunohistochemistry, performed as described (Miller et al., 2017). Primary antibodies used were: anti‐EGFP (rabbit, Life Technologies, 1:500; or mouse IgG1, Roche, 1:500); anti‐Elavl3/Elavl4 (anti‐HuC/D; mouse IgG2b, Invitrogen, 1:400); anti‐Tubb3 (neuronal class III beta‐tubulin; clone TUJ1, mouse IgG2a, Covance, 1:500). AlexaFluor‐conjugated secondary antibodies were obtained from Invitrogen.

### 4.6 Image capture and processing

Images were captured using a QImaging Retiga 2000R camera and an RGB pancake (QImaging) on a Zeiss AxioSkop 2 MOT compound microscope with QCapture Pro 6.0 software. Images were processed using Adobe Photoshop CS5.1 or CS6.

### 4.7 Statistical analysis

Initial data analysis was performed using Microsoft Excel. Scatter plots were generated using GraphPad Prism 7 (GraphPad Software, La Jolla, CA), which was also used to check all datasets for normality using the Shapiro‐Wilk test, and to compare variances using an *F* test. Means were compared in GraphPad Prism 7 using an unpaired two‐tailed Student's *t*‐test.

## COMPETING INTERESTS

The authors declare no competing or financial interests.
